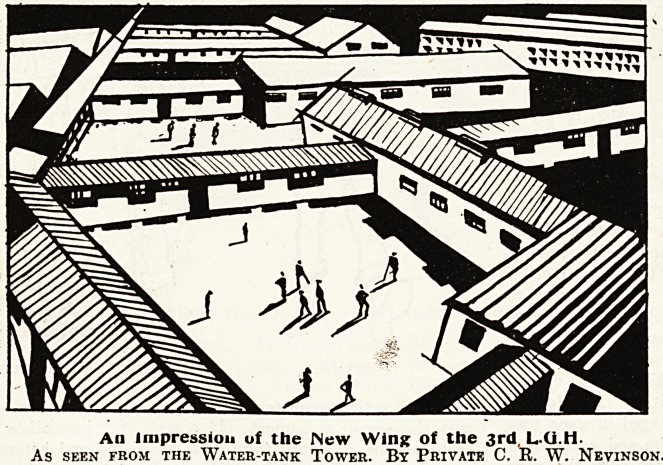# Every Hospital's Own Gazette: A Great Success at the Military Hospitals

**Published:** 1915-12-18

**Authors:** 


### EVERY HOSPITAL'S OWN GAZETTE.

### A Great Success at the Military Hospitals.

The military hospital magazine is something quite new in journalism. It is a product, and a very pleasing one, of the present war, emerging from the hurly-burly of the battlefield and associated with the aftermath of probably the most terrible struggle since the world began. It therefore naturally excites comparison with the hospital magazines to which we have long been accustomed, and, as we turn the lively pages of the periodicals now being issued from the military hospitals, we are at once struck with a liveliness, a journalistic ability, a public interest, and a high standard of illustration which seems to put the hospital magazine of peace-time completely into the shade. It is well, therefore, to reconsider what the hospital magazine ought to be, and is capable of being; for a moment's thought shows us that the *Gazettes* issued from the great hospitals and the new military hospitals are different—not merely that one, from the point of view of the general public, is dull, and the other entertaining, but that the real difference is rather a difference of kind than of degree. What, then, should a hospital magazine aim at?

It should aim at enlisting the interest and enthusiasm of the people in the area which it serves by spreading among them the spirit of hospital life which comes from acquaintance with its inner life and affairs. All classes resident in the neighbourhood of the hospital should be encouraged to feel a personal interest in it, and this personal interest is best fostered by knowledge of its doings, its atmosphere, its needs, and the nature of its work. The hospital should be the common bond which unites members of every other organisation, holders of every shade of opinion, all classes in its area. Its affairs should be known to everybody. Its atmosphere should be everywhere appreciated, and while these results can only be gained by everyone in some way coming into contact and relation with the institution, one of the best means of securing intimacy and maintaining interest is by a well-written hospital magazine. Those who visit the hospital will naturally turn to its pages; those who do not should be led by the information diffused, and even by the amusement provided, to do so. For by making the hospital everybody's care and pride, work on its behalf comes rightly to be regarded as the most acceptable and honourable in which any public-spirited citizen can be engaged.

The above has only to be read by readers acquainted with the customary civil hospital *Gazette* for them to realise that whatever the old *Gazette* has aimed at it has not aimed at anything like this! In point of fact, of course, the hospitals with medical schools have had *Gazettes* for their medical students, which have catered for the school, and the school alone, chronicled the school's successes, recorded appointments, some current gossip, some fragmentary correspondence, a joke or two, and, probably, included an address or lecture of a technical medical kind.

### A Type of What Can be Done.

The hospital magazine now being produced by the military hospitals, which we have called above a new feature in journalism, is a totally different conception, and covers vastly wider ground and interests. Indeed, the new magazines are remarkable for their liveliness, general interest, and illustrations. They are still called *Gazettes*, but magazines would be a better name for them. Let us illustrate their scope by an example.

Of such *Gazettes* there is more than one, but without undervaluing the merit of any other it can, we think, be said that the *Gazette* of the 3rd London General Hospital is a unique production. The first number, which was published in October, attracted considerable public attention, for, although it was produced mainly for the pleasure of the sick and wounded under treatment at Wandsworth, and for the entertainment of the staff, its merits were soon discovered outside the walls of the hospital, and the demand for copies was far in excess of anything the editor had expected or for which the publishers were prepared. To give some idea of what this *Gazette* is like and how it is produced is the object of this article. To

*Photo by*] [*Elliott and Fry, Ltd.*
Private Ward Muir,
Editor of the *Gazette*, 3rd L.G.H., Wandsworth.

begin with, it should be stated that practically all the articles, verses, and drawings are by members of the unit from the commanding officer and principal matron downwards or by soldiers under treatment. Exceptions to this rule are few, but there are exceptions—for instance, to the Christmas Number, which has just made its appearance, some verses have been contributed by Miss Ella Wheeler Wilcox—but, broadly, it may be said that the editor finds all his contributors on the premises.

### A Staff of Artists.

Before producing a paper it is necessary to find an editor, and fortunately this was an easy task at Wandsworth, for among the orderlies was one Private Ward Muir, who in civil life is a distinguished journalist, but has for the time being laid down his pen in order to serve his country in another way. If the hospital was fortunate in finding so experienced a journalist for the post of editor, the editor was equally fortunate in finding close at hand a staff of artists which even our best illustrated papers ought to find all-sufficient. So well is this art represented at the hospital that the new recreation room was opened in the early autumn with an exhibition of pictures and sculpture, the work of artists serving with this unit. The exhibition was visited by Princess Louise (Duchess of Argyll), Princess Henry of Battenberg, and Princess Marie Louise of Schleswig-Holstein. The exhibitors were Private Bowcher, Lance-Corporal Coates, Corporal Craft, Private de la Bere, Sergeant Evergood, Private Fagan, Corporal Fullwood, Private Grant, Private Hampton, Private Irving, Private Kirk, Private Lee, Private Lobley, Private Martin, Lance-Corporal Mulock, Private Nevinson, Private Paget, Private Pirie, Lance-Corporal Roberts, Private Shewring, Private Stott, Lance-Corporal Streeton, Private Ware, Private Wilcoxson, Sergeant Withers, and Sergeant Derwent Wood.

**The Light that Failed. By Lance-Corporal Geo. J. Coates.**

### Why so Many Artists?

One may well ask how it came about that so many artists are to be found among the staff, and this is the explanation: Mr. Alfred Withers, who is now Sergeant Withers, joined the unit as soon as war broke out. Later, when it was found that more men were required, he suggested that many artists would probably like to join them if they were invited to do so. Lieutenant-Colonel Bruce Porter, the commanding officer, who regarded the suggestion as a good one, did the best possible thing under the circumstances; that is to say, accompanied by Sergeant Withers, he paid a visit to the Chelsea Arts Club, with the result that some twenty artists promptly answered the call and were enrolled as members of the unit. Among them was Mr. Derwent Wood, A.R.A., who is now a sergeant and is in charge of the plaster-splint workshop. With all this talent at his disposal the work of Private Ward Muir in providing pictures for his paper was not extraordinarily difficult. But it should be remembered that the orderlies and non-commissioned officers forming part of a military hospital unit have very little time for pursuing occupations outside their hospital duties; after the day's or the night's work is done they are not much disposed for work of another kind, and only a labour of love would entice them from their well-earned rest and recreation. By the generous permission of the editor of the *Gazette* we are able to produce some excellent examples of the artistic productions which adorn the pages of the *Gazette* month by month. At this hospital the task of finding illustrators is less difficult than that of finding writers—surely a reversal of the normal position.

### The Scarcity of Article Writers.

Poets are seldom difficult to find if one is not particular as to the quality, thus the editor tells us in his first number "we little guessed, when we advertised for literary contributions, how prompt would be the response from poets. While the artists and article writers had to be goaded to submit material for the magazine, the poets needed no second invitation; they burst into song (so to speak) on every hand, and their lyrics soon littered the editorial table." That some of these were good is evident from the fact that they have passed a discriminating editor and found their way into print. Thus Miss D. Eardley-Wilmot, whose song "A Little Grey Home in the West" has been heard at many a concert in the recreation room—and in the trenches too—and who was a nurse on night duty in Ward C 3, contributed to the November Number some verses, of which these are two:

C 8 has got a poet, you can feel it in the ward,
But, oh! she'd like to drop the muse and take up with the sword,
For the art of writing verses may be quite a pretty gift,
But it doesn't lend a man a hand, or give a man a lift.

She has always preached against it as a very useless thing,
But somehow, hidden in her, there's a lark that's got to sing!
And if writing verse on duty is a criminal offence—
Well, she just has got to write them, and that's all for her defence.

Some Sentry Stories.

To the same number Miss Nightingale, who is distantly connected with the great Florence Nightingale, and is one

When East meets West; a Sketch in the Grounds.
Misfits will happen, even in the best regulated hospitals.
Both in Blue: Shall we go to the clothing store, or shall we swop right here?

of the hospital's "postmen," contributed some verses on "The Men In Blue," and in the previous issue she related some of her experiences as a "postman." Of articles in lighter vein there are many, and some of the jokes are distinctly good, especially the sentry stories, as, for instance, this one: "A sentry who was new to the regiment had been moistening earth's poor clay to an undue extent. When the colonel passed he duly challenged him, and was told very sharply, 'I am the colonel.' He confidently remarked, 'You got a dashed good job, don't get drunk and lose it.'" The following is another: "A young recruit managed in some way to rough up his colonel, who sent for him and gave him a rare jacketing, then put him on sentry duty the next day. The young 'un was still out of luck, and was hauled up again by the colonel, who angrily asked, 'Why the devil didn't you salute when I passed just now?' And here was the innocent reply, 'I thought you were still cross with me.' Isn't that truly lovely and natural?" When we mention that the Commanding Officer, the Principal Matron, and the Matron have written for the *Gazette*, we need hardly say that space is devoted to more serious contributions than those to which we have just referred.

The Power of Success.

Here, then, is a proof of what can be done. It remains to consider how the evident success has been accomplished. Probably the first cause is simply that spirit of comradeship which is always strongly aroused at the start of a new institution that is staffed by volunteers. Everyone is a beginner, and everyone is an enthusiast. It is all an adventure; trouble, discipline, discomfort, all are something of a joke. It is for no one a question of settling down into a groove that will permanently solve the difficult problem of getting a living. Then, of course, the success of the hospital magazine has been largely due to the presence on the new staffs of artists and journalists of experience who, along with men of all classes, have joined the units. For instance:

A good idea of the catholicity of this war, and incidentally some sort of clue to the success of the above paper, can be obtained from a statement as to the variety of professions and trades from which the R.A.M.C. (T.) unit of the 3rd L.G.H. has been recruited. Amongst the orderlies there are several schoolmasters, two actors, a lexicographer, two dentists, a cinema pianist, a piano-tuner, a fireman, a novelist, a retired professional boxer, a racehorse trainer, a barrister, a mining engineer, a member of the Stock Exchange, a character vocalist, a stage carpenter, a Tube liftman, as well as the artists to whom we have already alluded. The unit is only recruited from men who have either been rejected (or certified

Specials.

A Sisterly Admonition.

unfit) for active combatant service, or who are under or over the age of enlistment. If a staff of "journalists" could not be produced from such promising and heterogeneous material then it could be produced from nowhere. Thrown together with a common purpose, and from such diverse professions and occupations, the whole institution, with its touch of adventure, becomes that of a kind of Mermaid Tavern. Wit and literary activity must result from such a mixture of men. Among the factors of the military hospital magazines' success we must also include the fact that the convoys received generally bring a crop of vitalising material, jokes and incidents from the Front, such as simply cannot be provided by ordinary civil life, as most hospital patients have known it. Then, again, the spirit of good fellowship and cheerfulness among the wounded seeks expression, and is caught up and reflected by the orderlies in articles, chaff, caricatures, and verse, in a way which has little, if any, existence in the case of the sick civilian patient. The magazine we have been considering provides a striking example of this truth, which is well worth quoting :

The following lines are by one whose identity is buried in the description "A 3rd L.G.H. Orderly" :

When the war is done we'll recall the fun—
The fun that conquered the pain—
For we'll owe a debt (and we'll not forget)
To the jokes that kept us sane :

How the wounded could laugh and bandy their chaff,
And kick up a deuce of a row!
. . . *It may be, in peace, when the sufferings cease,*
*We'll be sadder, aye sadder, than now.*

There you have, in the guise of light verse, a genuine factor of the life in a military hospital. Can anyone doubt its value to the hospital magazine? How is it possible for the ordinary hospital to vie, in journalistic success, with a product evolved in such a spirit?

It should be added that the movement which has produced the hospital magazine has spread also to Ireland, and has there adopted the more definitely public appeal of a Christmas Book and Calendar, with the deliberate design of awakening public interest. For instance,

**An Impression of the New Wing of the 3rd L.G.H.**
AS SEEN FROM THE WATER-TANK TOWER. BY PRIVATE C. R. W. NEVINSON.

and it is a capital example, the Ulster Volunteer Force Hospital has produced an astonishingly able Book or Calendar, designed to hang on the wall, with illustrations to every page, and really apt quotations beneath them. Sir E. Carson is the hero of the Book, which opens, after a good portrait of His Majesty, with a message reproduced from Sir Edward's handwriting, and proceeds, page after page, to unfold the growth and history of the hospital, with capital photographs of every phase of its existence. These photographs have been chosen

DAWN-JULY 1915

**Dawn in a Hospital Ward. Sketch by Private Paul Kirk.**

and arranged with evident care, and the whole forms a model appeal to the general public, though, of course, it lacks the intimate personal touches of artistic skill, humour, and pathos which make the military hospital magazine the living thing which it is. We mention the Christmas Book of the Ulster Volunteer Force Hospital in the same breath as the magazine of the 3rd L.G.H., Wandsworth, because the ideal hospital magazine of the ordinary hospital in peace-time should be a combination of the two.

We believe that in spite of difficulties in securing this combination much can be done. For the lesson of all this must be to encourage hospital managers to apprehend the mass of good material which surrounds them in the national crisis. If a tithe of it only were made use of by the medium of hospital magazines the popularity of the hospitals might be still enormously extended in such a way as to recruit a multitude of workers to continue, in peace as well as in war, to rally round the institutions, to maintain the impulse of gratitude engendered by the hospitals' work for the wounded and to give the whole voluntary system a vastly extended influence, as well as to guarantee its future on an even more ambitious scale. Here is the opportunity which the hospital magazine might powerfully aid, if only the editors could be found—the men, that is, with an eye to see what is really going on around them. To draw attention to the possibilities of this means of extending our hospitals' influence we have shown in detail by one example how much can be done.

It remains for the men in the hospitals to take up the idea here illustrated and to act on it.

## Figures and Tables

**Figure f1:**
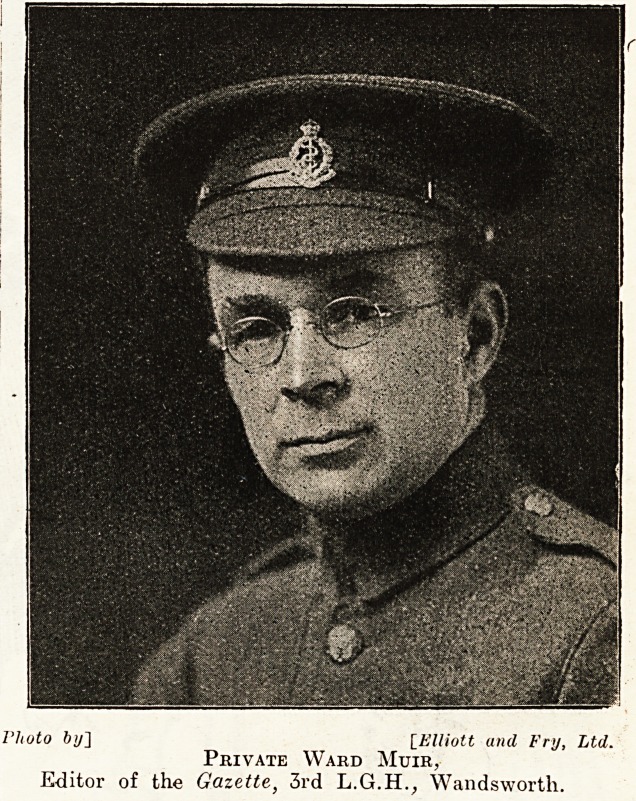


**Figure f2:**
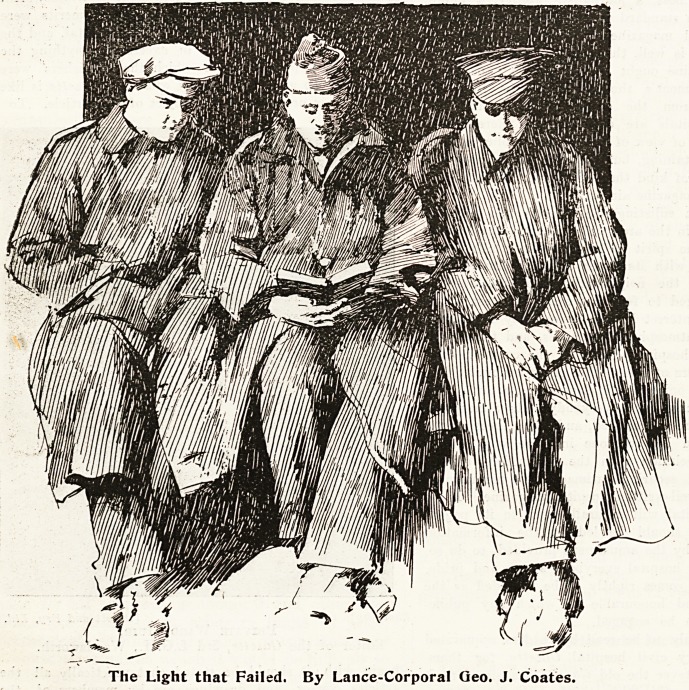


**Figure f3:**
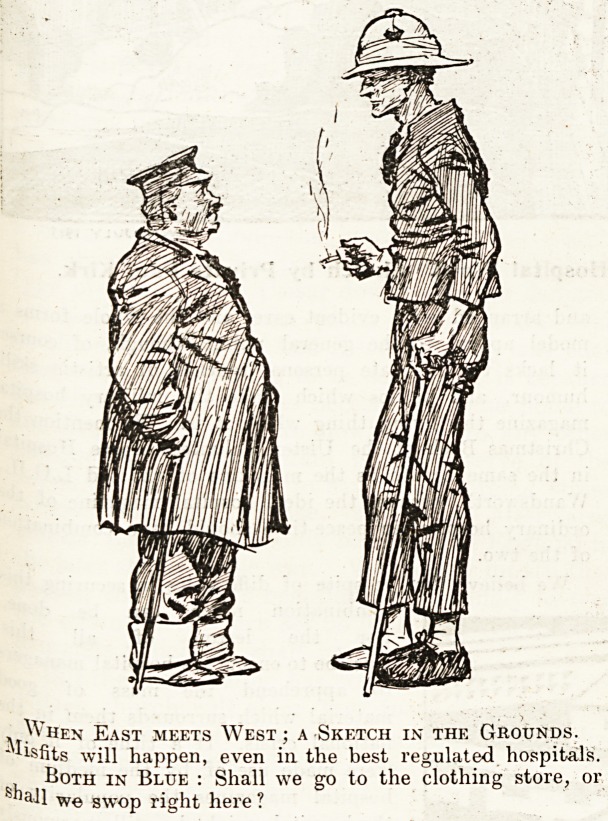


**Figure f4:**
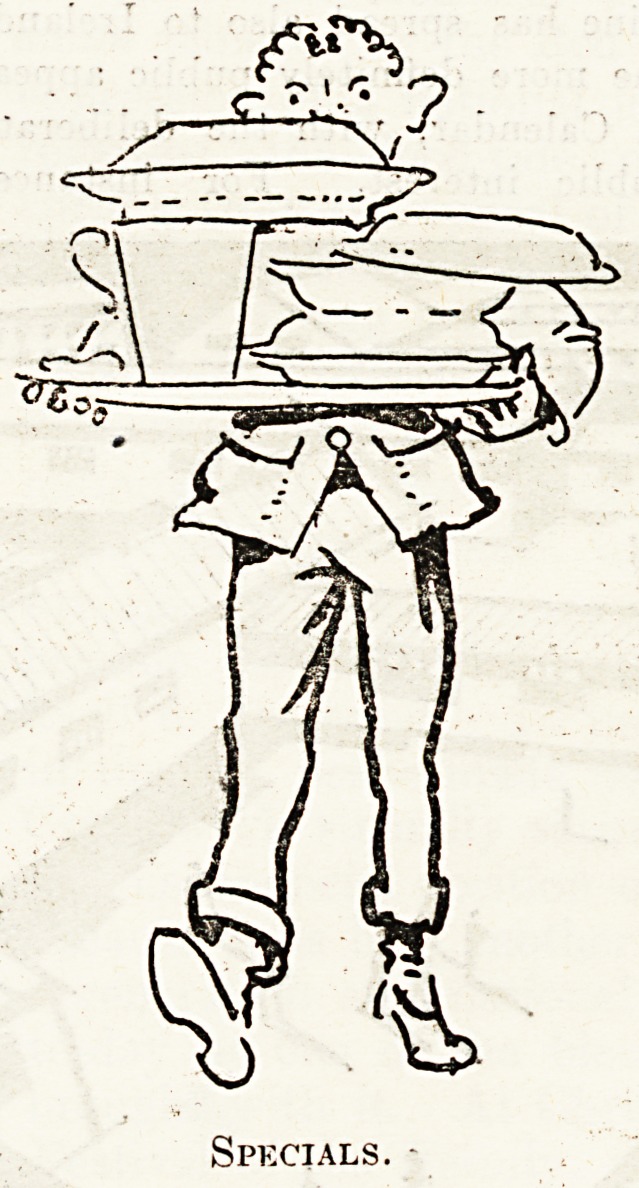


**Figure f5:**
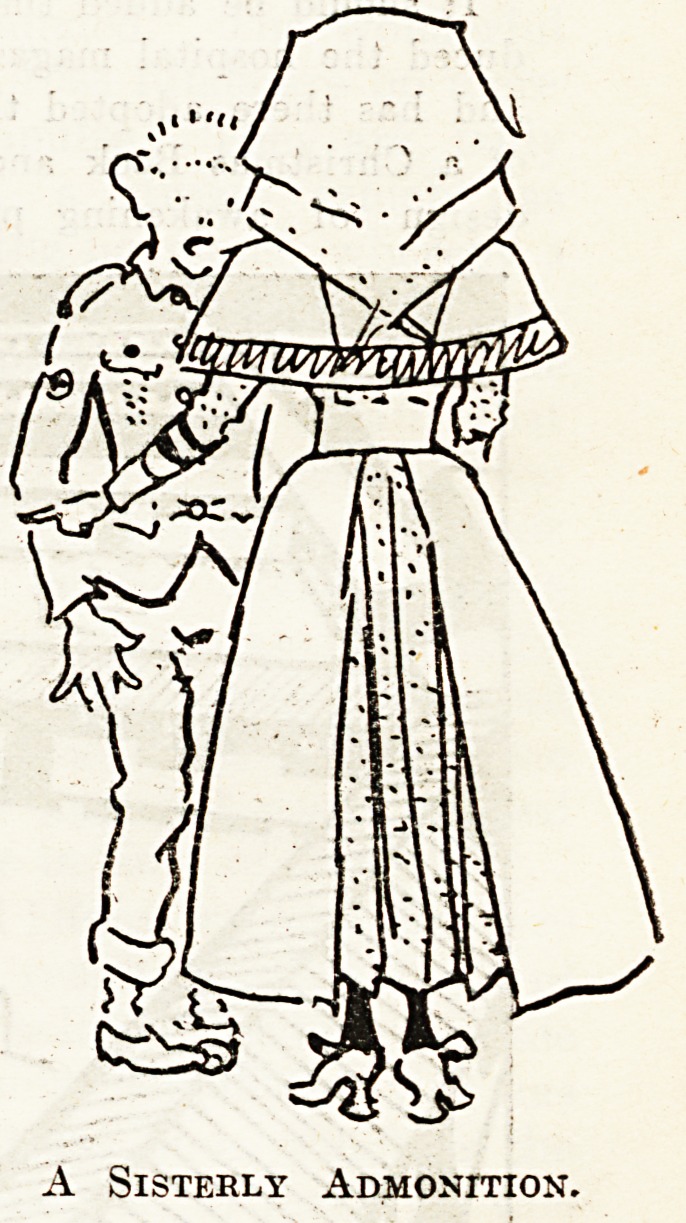


**Figure f6:**
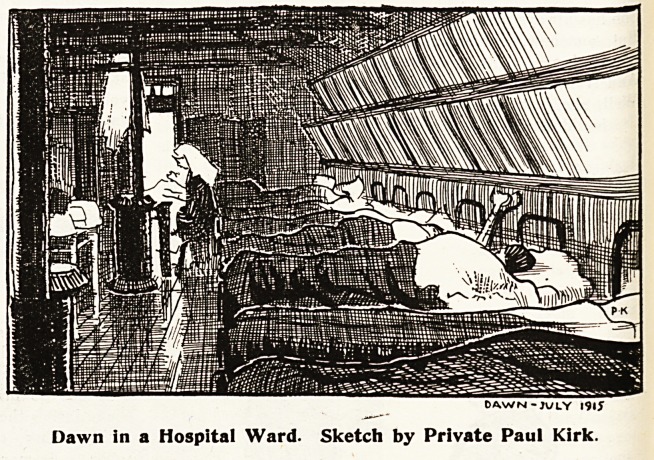


**Figure f7:**